# Supplementary material for: Corynebacterium striatum—Got Worse by a Pandemic?
Source: Pathogens. 2022 Jun 14;11(6):685. doi: 10.3390/pathogens11060685 (PMC9230073; doi:10.3390/pathogens11060685)
Supplement: Supplementary file 1 [file pathogens-11-00685-s001.zip › pathogens-1761169-supplementary.pdf]

Pycaret-based prediction of resistance of C. straitum strains from 2022 until 2030:

Ciprofloxacin:
Model: Croston
α=0.02445940685773727

Moxifloxacin:
Model: NaiveForecaster
Seasonal periodicity=1
Strategy='last'
Window_length=None

Clindamycin
Model: Croston
α=0.02079310220576088

Gentamicin:
Model: AutoARIMA
D=None, alpha=0.05, d=None, error_action='warn', information_criterion='aic', D=1, d=2, ma x_order=5, maxiter=50, method='lbfgs', n_fits=10, n_jobs=1, offset_test_args=None, out_of_ sample_size=0, random=False, scoring='mse', scoring_args=None, seasonal=True, seasonal_t est='ocsb', seasonal_test_args=None, sp=1, P=1, Q=1, p=2, start_params=None

Rifampicin:
Model: BaseCdsDtForecaster
degree=1, deseasonal_model='additive', regressor=HuberRegressor(alpha=0.0001, epsilon=1 . 35, fit_intercept=True, max_iter=100, tol=1e-05, warm_start=False), sp=1, window_length=1

Tetraciklin:
Model: Croston
α=0.1

Linezolid:
Model:BaseCdsDtForecaster
degree=1, deseasonal_model='additive', regressor=PassiveAggressiveRegressor(C=1.0, avera ge=False, early_stopping=False, epsilon=0.1, fit_intercept=True, loss='epsilon_insensitive', m ax_iter=1000, n_iter_no_change=5, random_state=123, shuffle=True, tol=0.001, validation_fr action=0.1, verbose=0, warm_start=False), sp=1, window_length=1)
